# Editorial: The Computational Analysis of Cultural Conflicts

**DOI:** 10.3389/fdata.2022.840584

**Published:** 2022-03-01

**Authors:** Petter Törnberg, Eckehard Olbrich, Justus Uitermark

**Affiliations:** ^1^Amsterdam Institute for Social Science Research, University of Amsterdam, Amsterdam, Netherlands; ^2^Max Planck Institute for Mathematics in the Sciences, Leipzig, Germany

**Keywords:** conflict, digital media, computational social science, natural language processing, polarization

## The Computational Analysis of Cultural Conflicts

Our society is currently facing a set of interrelated crises – the climate crisis, the inequality crisis, and a crisis of political legitimacy – as well as growing political polarization and ethnic and cultural conflict. These growing crises begs questions of how we manage the unanticipated side effects of our technological innovation. Our capacity to respond to these crises itself appears to be in a state of crisis, as the media and communication technology through which we mobilize our response are designed not to aid agreement, but to maximize user engagement by intensifying controversy and conflict (Törnberg and Uitermark, 2020). At a time when we most direly need to come together in constructive alignment to face these growing crises, we have created a public sphere with a bias for the conflictual, the sensational, and the hyper-partisan, shifting discourse in ways that reduce our collective ability to constructively respond (Rogers in this Research Topic; Törnberg, 2018; Gaisbauer et al., 2020; Rogers, 2020; Törnberg et al., 2021).

As Global Warming is paralleled by rising temperatures in our political climate, we must ask how to manage the technology that determines our ability to engage in constructive collective political action. Heeding the calls to become global stewards for a sustainable environment requires first becoming stewards of the technology that shape our collective behavior – and for that, we must understand the complex relationship between media technology and cultural conflicts (Törnberg and Uitermark, 2021).

This question is the focus of this Research Topic. The contributions to the collection come out of the ODYCCEUS research project, an EU-funded research project, subsidized in the context of the FET Proactive funding scheme. ODYCCEUS brought together scholars in the natural sciences (physicists, computer scientists, and mathematicians) and the social sciences and humanities (sociologists, geographers, and media scholars) to develop new methods and tools for the study of cultural conflict.

This project asked whether and how the ever-growing technological capacities of our computational tools and digital data can be reimagined and leveraged to understand and resolve conflicts: How are our communication technologies implicated in conflict? Can they help us understand and resolve our conflicts and disagreements? Can they help match our ability to change our world with our ability to understand ourselves and orient our collective capacity for action?

Answering these questions in turn requires interrogating and expanding the scope of Computational Social Science: How do we incorporate meaning-making processes that are central to cultural life? How do we account for power and inequalities? How can we account for reflexivity? And how can we grasp qualitative change? How do we account for the impact of digital platforms in shaping the cultural life and conflicts that takes place on them?

This special issue showcases some of the project's work on applying computational methods and digital data to examine conflicts, and deal with the societal challenges we face.

Levis Sullam et al. do so by focusing on the processes through which we construct and dehumanize an Other, using computational text analysis to study the shifting associations and stereotypes around Jews during to the fin-de-siècle France's “Dreyfus Affair”– which Hannah Arendt famously referred to as a “dress rehearsal” for the Holocaust. Grasland et al. similarly employ computational methods to study the representation of various kinds of crises in international press. This work shows how these digital methods can be used to reveal the cultural processes laying the ground for conflict. The authors develop an observatory that allows users to track media reports over time and across space. Their findings suggest quantitative and qualitative differences in how crises are discussed between different countries.

Similar discursive shifts are the focus of Peeters et al.'s examination of word play and toxic memes on the online 4Chan community. This work shows the role of the digital platforms in co-shaping the subcultural dynamics that were part of the rise of recent wave of white supremacism. Rogers focuses specifically on this question: how do media platforms filter and reshape news according to certain biases? The study found that TikTok, Twitter, 4chan, Reddit, Facebook, and Google all bring their own biases that shape how we understand the world. Pointing toward a hopeful alternative to this biased representation, founded in a causal ontology, Willaert et al. develop methods that may help make sense and expose conflicting conceptualization of the growing wealth of information about the world. Focusing on news articles on climate change, this method parses the causal claims of the text, and uses these to present readers with competing causal statements.

Van Vliet's contribution however suggests that conflicts may persist even when these causal claims are made explicit: focusing on the Brexit debate, this work shows that political conflicts can be rooted in contradictory fundamental values, as morality becomes entrenched within political positions. As computational tools can throw light on such differing values, we may hope to find more constructive ways of channeling such value disagreement.

Olbrich and Banisch focus on applying textual analysis to face the question of our political system's capacity to channel conflicts into the political system. Using party manifestos, they show how computational methods can be used to translate these texts into spaces of disagreement, in which shifts such as globalization produce new conflict dimensions that come to reorient party politics.

Together, these studies take important steps toward reorienting computational methods and digital data toward the study of cultural conflicts, and facing the challenging epistemic question involved in doing so. This work may help shift not only our scientific understanding, but also play a part in establishing a new foundation for the digital methods that we employ in our daily lives, creating new communication technologies that may support our constructive resolution of disagreements, and collective alignment to face our common challenges.

## Author Contributions

PT wrote the first draft of the manuscript. JU and EO discussed and revised the manuscript. All authors contributed to the manuscript revision, read, and approved the submitted version.

## Funding

This project has received funding from the European Union's Horizon 2020 research and innovation programme project ODYCCEUS (Grant Agreement No. 732942). The funders had no role in study design, data collection and analysis, decision to publish, or preparation of the manuscript.

## Conflict of Interest

The authors declare that the research was conducted in the absence of any commercial or financial relationships that could be construed as a potential conflict of interest.

## Publisher's Note

All claims expressed in this article are solely those of the authors and do not necessarily represent those of their affiliated organizations, or those of the publisher, the editors and the reviewers. Any product that may be evaluated in this article, or claim that may be made by its manufacturer, is not guaranteed or endorsed by the publisher.
